# Combination immunotherapy targeting LAG-3, PD-1 and STING suppresses hepatocellular carcinoma as monitored by LAG-3 targeted PET imaging

**DOI:** 10.1186/s40364-025-00820-z

**Published:** 2025-08-12

**Authors:** Zhen Quan, Yu Gao, Bo Sun, Yiwan Guo, Ziwei Jin, Na Hao, Dawei Jiang, Chuansheng Zheng, Xin Li, Quan Chen

**Affiliations:** 1 Department of Radiology, Union Hospital, Tongji Medical College, Huazhong University of Science and Technology, Jiefang Avenue #1277, Wuhan, 430022 China; 2 Hubei Provincial Clinical Research Center for Precision Radiology & Interventional Medicine, Wuhan, 430022 China; 3 Hubei Key Laboratory of Molecular Imaging, No. 1277 Jiefang Avenue, Wuhan, 430022 Hubei China; 4 Department of Nuclear Medicine, Union Hospital, Tongji Medical College, Huazhong University of Science and Technology, Wuhan, China

**Keywords:** Programmed cell death protein 1 (PD-1), Stimulator of interferon genes (STING), Lymphocyte activation gene 3 (LAG-3), PET imaging, Combination immunotherapy

## Abstract

**Background:**

The low response rate of anti-PD-1 monoclonal antibodies (mAbs) in hepatocellular carcinoma (HCC) requires the development of combination immunotherapy strategies to improve their efficacy. This study aimed to use LAG-3-targeted PET imaging to monitor the efficacy of anti-PD-1 mAb, a stimulator of interferon genes (STING) agonist, and anti-LAG-3 mAb, both individually and in combination. Furthermore, we evaluated the potential of a triple immunotherapy regimen (anti-PD-1 mAb, STING agonist, and anti-LAG-3 mAb) to improve HCC treatment.

**Methods:**

The LAG-3 inhibitor C25 based on a cyclic peptide was chelated with NOTA, radiolabeled with [^68^Ga]GaCl_3_. The resulting [^68^Ga]Ga-NOTA-C25 underwent in vivo PET imaging and ex vivo biodistribution examination in Hepa1-6 tumor-bearing mice. [^68^Ga]Ga-NOTA-C25 PET was used to monitor the efficacy of monotherapy and dual immunotherapy with anti-PD-1 monoclonal antibody (mAb) and STING agonists. The tumor uptake of [^68^Ga]Ga-NOTA-C25, tumor response, and survival rates were measured following different treatments. The therapeutic efficacy, molecular mechanisms, and safety of triple immunotherapy were validated using histopathological analysis and flow cytometry.

**Results:**

[^68^Ga]Ga-NOTA-C25 PET imaging effectively and noninvasively detected LAG-3^+^ tumor-infiltrating lymphocytes (TILs) in Hepa1-6 tumor-bearing mice. In mice treated with anti-PD-1 mAb, STING agonist, or a combination immunotherapy, [^68^Ga]Ga-NOTA-C25 PET revealed significantly increased LAG-3^+^ TIL levels. At the treatment endpoint, the combination of the STING agonist with the anti-PD-1 mAb resulted in a significantly higher uptake (1.35 ± 0.191%ID/g) compared to the control group (0.402 ± 0.017%ID/g), the anti-PD-1 mAb group (0.647 ± 0.037%ID/g), and the STING agonist group (0.874 ± 0.089%ID/g). Uptake of [^68^Ga]Ga-NOTA-C25 was positively correlated with tumor therapeutic effects and survival rates. Triple immunotherapy with anti-PD-1 mAb, a STING agonist, and anti-LAG-3 mAb further enhanced efficacy compared to any dual immunotherapy regimen, and treatment efficacy was linearly associated with [^68^Ga]Ga-NOTA-C25 tumor uptake.

**Conclusions:**

Anti-PD-1 mAb and STING agonists have shown notable synergy in upregulating LAG-3 expression on TILs in HCC, which can be successfully tracked by [^68^Ga]Ga-NOTA-C25 PET imaging. Furthermore, integration of a triple immunotherapy regimen comprising an anti-PD-1 mAb, STING agonist, and anti-LAG-3 mAb demonstrated a significant improvement in therapeutic efficacy over dual immunotherapy approaches.

**Supplementary Information:**

The online version contains supplementary material available at 10.1186/s40364-025-00820-z.

## Introduction

Liver cancer is the sixth most common cancer and the third leading cause of cancer-related death worldwide [1]. Recent advances in immune checkpoint inhibitors (ICIs), including programmed cell death protein 1 (PD-1)/programmed death ligand 1 (PD-L1) and cytotoxic T lymphocyte-associated antigen 4 (CTLA-4), have shown promise for improving the prognosis of patients with hepatocellular carcinoma (HCC). The US Food and Drug Administration (FDA) has approved anti-PD-1 monoclonal antibodies (mAbs) nivolumab and pembrolizumab as second-line therapies for advanced HCC. However, the objective response rate of ICI monotherapy in HCC remains low at only 15%, and at least 30% of patients with HCC develop resistance to PD-1 inhibitors [2, 3]. The immunophenotypes of tumors can be broadly categorized into four types based on the density of immune cell infiltration within the tumor microenvironment (TME), which are crucial determinants of prognosis and response rates to ICIs immunotherapy [4]. Specifically, “hot” tumors are characterized by high T-cell infiltration, while “non-hot” tumors are subdivided into three groups: “cold” tumors absence of T-cell infiltration, “immunosuppressive” tumors with intermediate T-cell infiltration, and “excluded” tumors where T cells are unable to efficiently infiltrate into the tumor [5].

Combination immunotherapy presents a promising strategy to improve response rates and convert tumor immunophenotypes from “non-hot” to “hot,” thus reducing drug resistance [6]. Dual combination therapies, such as ICIs paired with antiangiogenic agents or a combination of two different ICIs, have shown promising results in HCC. However, these approaches have limited benefits for overall survival (OS). Triple immunotherapy has the potential to reduce resistance to ICI, offering a more effective approach to overcome treatment challenges and improve outcomes in HCC [7]. Early prediction of immunotherapy efficacy and monitoring of immune responses are crucial to improve clinical outcomes. However, currently there is a lack of effective biomarkers to guide combination immunotherapy strategies, which remains one of the top 10 challenges in cancer immunotherapy [8–10].

As the efficacy of PD-1 inhibitors in HCC is unsatisfactory due to primary or acquired resistance, researchers have gradually shifted their focus to novel ICIs. Lymphocyte activation gene 3 (LAG-3) is a promising immune checkpoint and is considered the third checkpoint after PD-1 and CTLA-4 [11, 12]. LAG-3 is a type I transmembrane protein expressed in various immune cells, including activated T cells, regulatory T cells (Tregs), B cells, and natural killer (NK) cells [13–15]. LAG-3 expression is often upregulated in tumor-infiltrating lymphocytes (TILs) of patients treated with anti-PD-1 mAbs [16]. Blocking the LAG-3 pathway can activate antigen-specific T cells within the TME, restore their cytotoxic function, and inhibit tumor growth. The combination of anti-LAG-3 mAb relatlimab and anti-PD-1 mAb nivolumab showed better progression-free survival compared to nivolumab monotherapy in melanoma [17]. Furthermore, high expression of LAG-3 in TILs has been found to be significantly associated with a poor prognosis for immunotherapy in patients with HCC and, therefore, has the potential to serve as a biomarker to evaluate therapeutic efficacy [18–20].

Positron emission tomography (PET) is a noninvasive technique used to detect changes in TILs during immunotherapy. It is currently used to help clinicians identify patients who may benefit from immunotherapy and monitor immune responses longitudinally and dynamically [21, 22]. In a previous study, the targeted LAG-3 PET probe [^68^Ga]Ga-NOTA-C25, developed from a low-molecular-weight cyclic peptide (CVPMTYRAC), demonstrated high molar activity, excellent in vitro stability, and hydrophilicity, showing strong potential for accurately detecting LAG-3 expression in tumor-infiltrating lymphocytes (TILs) and evaluating the therapeutic efficacy of ICIs [23, 24].

To date, the therapeutic effects of LAG-3 inhibitors in patients with HCC have not been reported. Meanwhile, combination immunotherapy strategies for HCC can induce antitumor immunity by activating the cyclic guanosine monophosphate–adenosine monophosphate (GMP-AMP) synthase (cGAS)/stimulator of interferon genes (STING) signaling pathway [25, 26]. STING agonist monotherapy or combination with anti-PD-1 mAb dual immunotherapy can significantly improve the antitumor immune response [27–29].

This study aimed to demonstrate the utility of [^68^Ga]Ga-NOTA-C25 PET imaging for detecting LAG-3 upregulation in TILs in Hepa1-6 tumor-bearing mice treated with anti-PD-1 mAb and STING agonists. Furthermore, we explored whether triple immunotherapy with anti-LAG-3 mAb, anti-PD-1 mAb, and STING agonists can significantly enhance antitumor immune responses, thus improving the efficacy of HCC immunotherapy.

## Materials and methods

### Study design

The PET probe [^68^Ga]Ga-NOTA-C25, which targets LAG-3, was radiosynthesized, and the study was conducted for three rounds with different purposes:

- **Imaging specificity validation**: Hepa1-6 tumor-bearing mice were randomly assigned to a non-blocking (direct injection of [^68^Ga]Ga-NOTA-C25) or C25-blocking group (co-injection of excess C25 peptide with [^68^Ga]Ga-NOTA-C25) to assess the specificity of [^68^Ga]Ga-NOTA-C25 for LAG-3^+^ TILs via in vivo PET imaging and ex vivo biodistribution.
- **Combination immunotherapy assessment**: The efficacy of anti-PD-1 mAb and STING agonist combination immunotherapy in Hepa1-6 tumor-bearing mice was evaluated. LAG-3^+^ TILs upregulation was monitored using [^68^Ga]Ga-NOTA-C25 PET/CT.
- **Triple immunotherapy evaluation**: We monitored the Hepa1-6 tumor-bearing mice receiving triple immunotherapy (anti-PD-1 mAb, STING agonist, and anti-LAG-3 mAb) and evaluated their antitumor immune responses, molecular mechanisms, and safety profiles.

### Construction of NOTA-C25 precursors

Cyclic peptides were modified with 1,4,7-triazacyclononane-1,4,7-triacetic acid (NOTA). The NOTA-C25 precursor (NOTA-CVPMTYRAC) was synthesized by Shanghai Science Peptide Biological Technology Co., Ltd. (Shanghai, China). The molecular weight was confirmed by matrix-assisted laser desorption ionization-time of flight (MALDI-TOF) mass spectrometry, and sample purity was verified by high-performance liquid chromatography (HPLC), ensuring a purity of over 98% which as certified by the manufacturer (Fig.S1).

### Radiosynthesis of [^68^Ga]Ga-NOTA-C25

[^68^Ga]GaCl_3_ was obtained from a [^68^Ge]Ge/[^68^Ga]Ga generator (Isotopen Technologien München AG) by elution with a 0.05 M HCl. In separate 2 mL centrifuge tubes, 300 µL of a 0.25 M sodium acetate solution, 50 µL of NOTA-C25, and 1000 µL of the obtained [^68^Ga]GaCl_3_ were added. After thorough mixing, the reaction mixture was heated at 95℃ for 15 min to form the product of the [^68^Ga]Ga-NOTA-C25 complex with a reaction pH of approximately 4–5. Finally, the reaction mixture was purified using a C18 cartridge. The radiochemical purity of [^68^Ga]Ga-NOTA-C25 was measured by radio-HPLC (Shimadzu CTO-20 A). The chromatographic conditions were as follows: reverse-phase C18 column, gradient elution with acetonitrile/water (0.1% TFA), the flow rate was set at 1.2 mL/min and detection via UV and radioactivity detectors.

### Cells and animal models

All animal studies were approved by the Animal Care Committee of the Union Hospital, Tongji Medical College, Huazhong University of Science and Technology. Female C57BL/6 mice (6−8 weeks old) were obtained from Wuhan Moubaili Biotechnology Co., Ltd. (Hubei, China). Hepa1-6 murine HCC cells (purchased from the American Type Culture Collection) were cultured at 37℃ with 5% CO_2_. To establish the tumor models, 5 × 10^6^ Hepa1-6 cells were subcutaneously injected into the right shoulder. Tumor volumes were measured with a caliper every alternate day and calculated as (length × width^2^)/2.

### [^68^Ga]Ga-NOTA-C25 PET/CT imaging and blocking experiment

[^68^Ga]Ga-NOTA-C25 PET/CT imaging was performed when tumor volume reached 200−300 mm^3^. Hepa1-6 tumor-bearing mice (*n* = 3) were intravenously injected with 7.4 MBq (200 µCi, 1 µg) of [^68^Ga]Ga-NOTA-C25 under isoflurane anesthesia. For the blocking experiment, the C25 peptide (500 µg in 100 µL) was co-injected with [^68^Ga]Ga-NOTA-C25 into Hepa1-6 tumor-bearing mice (*n* = 3). PET/CT images were obtained in static mode for 5 min, followed by a CT scan in the fast mode using the TransPET Discoverist 180 system at 30, 60, and 120 min after injection of [^68^Ga]Ga-NOTA-C25 per mouse.

PET scans and imaging analyses were performed using a TransPET Discoverist 180 system (Raycan Technology Co., Ltd., Suzhou, China). PET images were reconstructed using the three-dimensional ordered-subsets expectation maximum (3D-OSEM) method with a voxel size of 0.5 × 0.5 × 0.5 mm^3^, while CT images were reconstructed using the FDK algorithm with 512 × 512 × 512 matrix. The images were displayed using Inveon Research Workplace software (Siemens Ltd., Germany). Quantitative PET was performed using the recorded data and the measured conversion factor. The uptake of [^68^Ga]Ga-NOTA-C25 in tumors and organs (including the liver, heart, and muscle) was obtained by drawing the region of interest and expressed as the percentage of dose injected per gram (%ID/g). Tumor-to-muscle (T/M), tumor-to-heart (T/H), and tumor-to-liver (T/L) ratios were calculated.

### Ex vivo biodistribution study

Hepa1-6 tumor-bearing mice were intravenously injected with 7.4 MBq (200 µCi, 1 µg) of [^68^Ga]Ga-NOTA-C25 and sacrificed at 30, 60, and 120 min (*n* = 3 per time point). For blocking studies, the C25 peptide (500 µg in 100 µL) was co-injected and the mice were sacrificed at 60 min (*n* = 3) [24, 30]. Blood, tumor, tumor-draining lymph nodes (TDLN), spleen, heart, liver, kidney, intestine, lung, femur, stomach, brain, skin and muscle tissue were collected and weighed. Radioactivity in tissues was measured using a gamma counter. The %ID/g values were calculated by adjusting for signal decay and normalizing to external [^68^Ga]Ga standards that were measured in triplicate.

### [^68^Ga]Ga-NOTA-C25 PET imaging monitoring anti-PD-1 mAb and STING agonist combination immunotherapy

When the tumor volume reached 100−150 mm^3^, Hepa1-6 tumor-bearing mice were randomly divided into four groups (*n* = 6): anti-PD-1 mAb (αPD-1, 200 µg, intraperitoneally, Bioxcell, Cat#BE0146), STING agonist (MSA-2, 25 mg/kg, orally, GLPBIO, Cat#129425-81-6), combination therapy (αPD-1 + STING), and phosphate-buffered saline (PBS) as the control group. The drugs were administered every 3 days (days 0, 3, 6, 9, 12). Tumor volume was measured every 3 days. Following previous approaches [24, 31], PET/CT imaging of tumor-bearing mice (*n* = 3) was performed on days 4 and 13. At the treatment endpoint, mice were sacrificed for the ex vivo biodistribution study, histopathological analysis, and flow cytometry.

### Monitor triple immunotherapy in Hepa1-6 tumor models

The procedures were consistent with previously described combination immunotherapy, and the therapeutic efficacy, molecular mechanisms, and safety of the triple immunotherapy (anti-PD-1 mAb, STING agonist, and anti-LAG-3 mAb) were validated using histopathological methods and flow cytometry. Mice with tumor volumes between 100 and 150 mm³ were randomly divided into four groups (*n* = 6): anti-LAG-3 mAb (αLAG-3, 200 µg, intraperitoneally, Bioxcell, Cat#BE0174), αLAG-3 + STING, αPD-1 + αLAG-3, and αPD-1 + STING + αLAG-3. Treatments were administered every 3 days (days 0, 3, 6, 9, and 12). Tumor volumes were measured every 3 days. At the treatment endpoint, mice were sacrificed, and samples were collected for histological evaluation and flow cytometry. Details on immunofluorescence staining, flow cytometry, and safety assessments are provided in the Supplementary Information.

### Survival analysis

Overall survival data for Hepa1-6 tumor-bearing mice (*n* = 8 per group) treated with different immunotherapy regimens (anti-PD-1 mAb, STING agonist, anti-LAG-3 mAb, monotherapy, or their combination) were recorded. Treatments were administered every 3 days (days 0, 3, 6, 9, and 12). Mice were sacrificed after reaching human endpoints (tumor volume ≥ 1500 mm^3^).

### Statistical analysis

All data were analyzed using the GraphPad Prism 9.0 software. Quantitative data were expressed as mean ± standard deviation (SD). One-way or two-way analysis of variance and unpaired two-tailed Student’s t-tests were used for statistical comparisons. One-way ANOVA with a post hoc Tukey’s test was used to compare multiple groups. The Kaplan-Meier survival curves were analyzed using the log-rank test. Pearson’s correlation tests were used for histological studies, flow cytometry, in vivo PET, and ex vivo biodistribution experiments. The *P*-value was set at 0.05.

## Results

### [^68^Ga]Ga-NOTA-C25 PET imaging can detect LAG-3^+^ TILs in a murine model of HCC

**Fig. 1:**
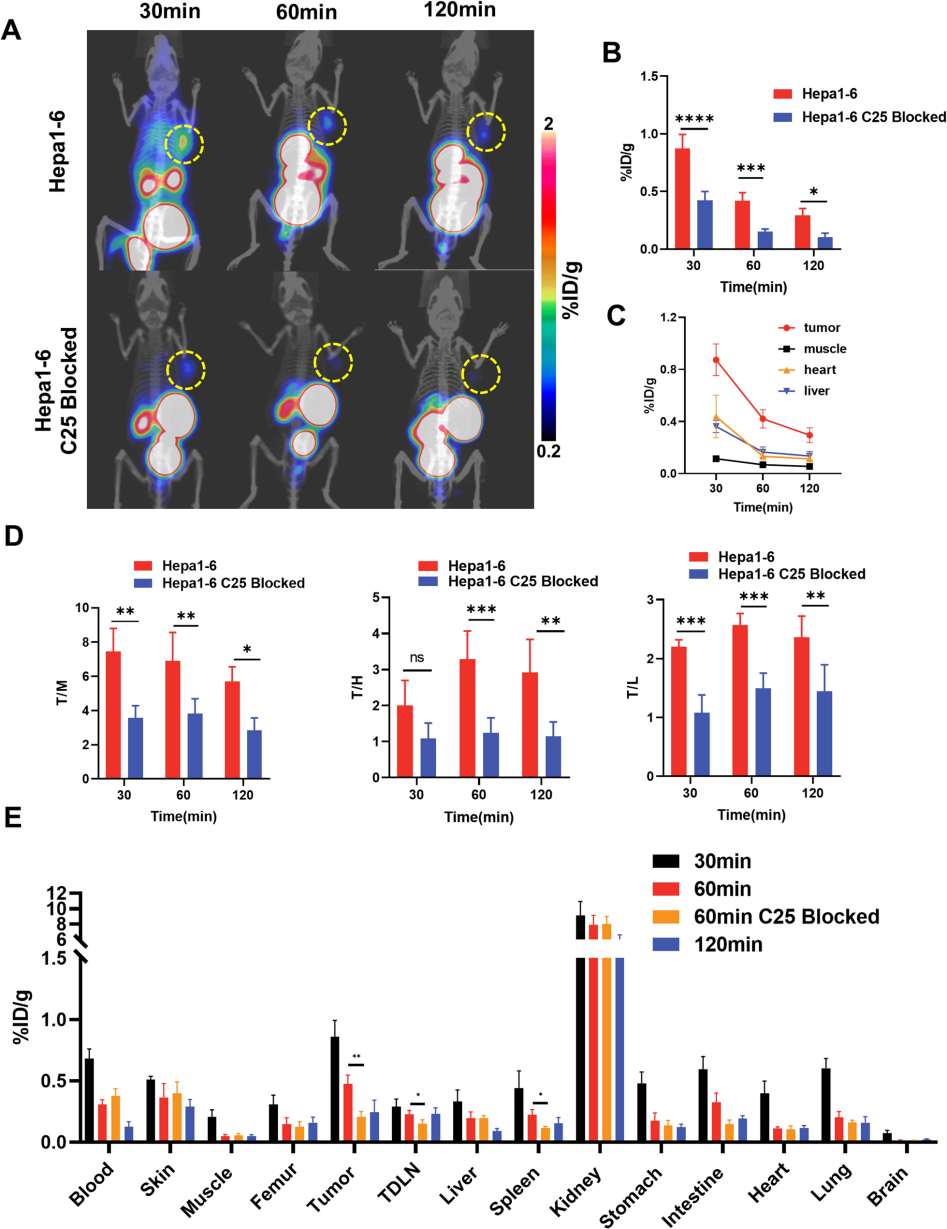
(**A**) PET/CT images of [^68^Ga]Ga-NOTA-C25 in Hepa1-6 tumor-bearing mice groups (yellow circles indicating tumors); (**B**) Comparison of [^68^Ga]Ga-NOTA-C25 uptake between non-blocking and blocking in Hepa1-6 tumors (*n* = 3); (**C**) Time-dependent uptake of [^68^Ga]Ga-NOTA-C25 in tumor (red), muscle (black), heart (orange), and liver (blue); (**D**) Tumor-to-muscle (T/M), tumor-to-heart (T/H) and tumor-to-liver (T/L) ratio at different time points after injection of [^68^Ga]Ga-NOTA-C25 (*n* = 3); (**E**) Ex vivo biodistribution of [^68^Ga]Ga-NOTA-C25 in Hepa1-6 tumor-bearing mice (*n* = 3). (*P < 0.05, **P < 0.01, *** *P* < 0.001, **** *P* < 0.0001)

[^68^Ga]Ga-NOTA-C25 was radiolabeled with high efficiency, resulting in a radiochemical purity of over 99% (Fig.S2) and a radiochemical yield exceeding 80%. PET imaging results demonstrated %ID/g of [^68^Ga]Ga-NOTA-C25 in Hepa1-6 tumors at 30, 60, and 120 min as 0.874 ± 0.121, 0.421 ± 0.070, and 0.296 ± 0.057, respectively. These values were significantly higher than those observed in the C25-blocked group (Fig. 1A and B). The tumor-to-background (T/B) ratio was favorable, as the uptake in the heart, liver, and muscle decreased over time and remained lower than that in the tumors (Fig. 1C). The tumor-to-heart (T/H) ratios at 60 and 120 min, but not at 30 min, were significantly higher in Hepa1-6 tumor-bearing mice than in the C25-blocked group (Fig. 1D). The tumor-to-muscle (T/M) ratio was significantly higher than that of the C25-blocked group at all time points (Fig. 1D). Optimal imaging performance was observed at 60 min, confirming the targeting efficacy of [^68^Ga]Ga-NOTA-C25 in Hepa1-6 tumor-bearing mice. These findings were consistent with the results of ex vivo biodistribution (Fig. 1E, Fig.S3A, Table S1).

### LAG-3 PET imaging can monitor the upregulation of LAG-3^+^ TILs induced by the STING agonist/anti-PD-1 mAb

**Fig. 2:**
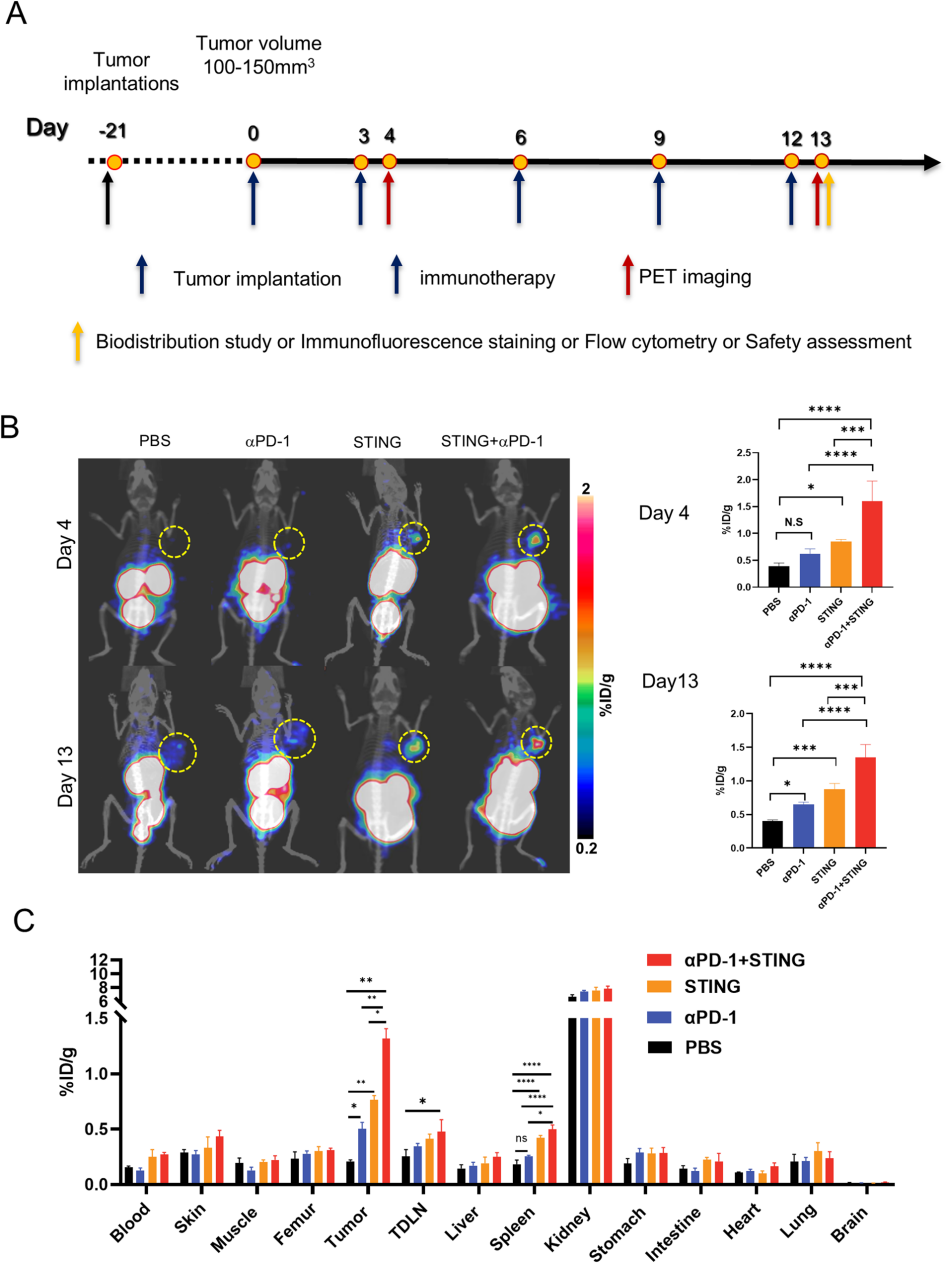
(**A**) Timeline of immunotherapy, PET imaging, biodistribution and ex vivo analysis in Hepa1-6 tumor-bearing mice; (**B**) Representative [^68^Ga]Ga-NOTA-C25 PET/CT images and quantitative analysis of Hepa1-6 tumor-bearing mice on day 4 and day 13 in different groups (yellow circles indicating tumors) (*n* = 3); (**C**) Ex vivo biodistribution of [^68^Ga]Ga-NOTA-C25 on day 13 (*n* = 3). (*P < 0.05, **P < 0.01, *** *P* < 0.001, **** *P* < 0.0001)

The effectiveness of [^68^Ga]Ga-NOTA-C25 PET imaging in detecting STING agonist/anti-PD-1 mAb induced upregulation of LAG-3^+^ TILs was assessed in a murine HCC model at early (day 4) and late (day 13) time points (Fig. 2A). On day 4, [^68^Ga]Ga-NOTA-C25 uptake was significantly higher in the STING (0.851 ± 0.037%ID/g) and STING + αPD-1 groups (1.60 ± 0.374%ID/g) compared to the PBS group (0.391 ± 0.061%ID/g). The tumor uptake of [^68^Ga]Ga-NOTA-C25 in the STING group was approximately two-fold higher than that in the PBS group, while the STING + αPD-1 group exhibited a three-fold increase compared to the PBS group. The tumor uptake of [^68^Ga]Ga-NOTA-C25 in the αPD-1 group (0.619 ± 0.095%ID/g) was elevated but not significantly different from the PBS group (Fig. 2B). Tumor uptake was highest in the STING + αPD-1 group, and this trend persisted consistently on days 4 and 13. On day 13, [^68^Ga]Ga-NOTA-C25 uptake remained significantly higher in the STING + αPD-1 group (1.35 ± 0.191%ID/g) compared to the PBS (0.402 ± 0.017%ID/g), αPD-1 (0.647 ± 0.037%ID/g), and STING (0.874 ± 0.089%ID/g) groups. Furthermore, a statistically significant difference in tumor uptake was observed between the αPD-1 group and the PBS group. No significant differences were found between day 4 and day 13 for the STING and STING + αPD-1 groups, indicating a sustained high level of immune activation (Fig. 2B). These results were corroborated by ex vivo biodistribution studies, which confirmed different upregulated tumor uptake of [^68^Ga]Ga-NOTA-C25 in the STING, αPD-1, and STING + αPD-1 groups compared to the PBS group (Fig. 2C, Fig.S3B, Table S2). In particular, the STING + αPD-1 group exhibited higher [^68^Ga]Ga-NOTA-C25 uptake compared to the STING and αPD-1 monotherapy group.

**Fig. 3:**
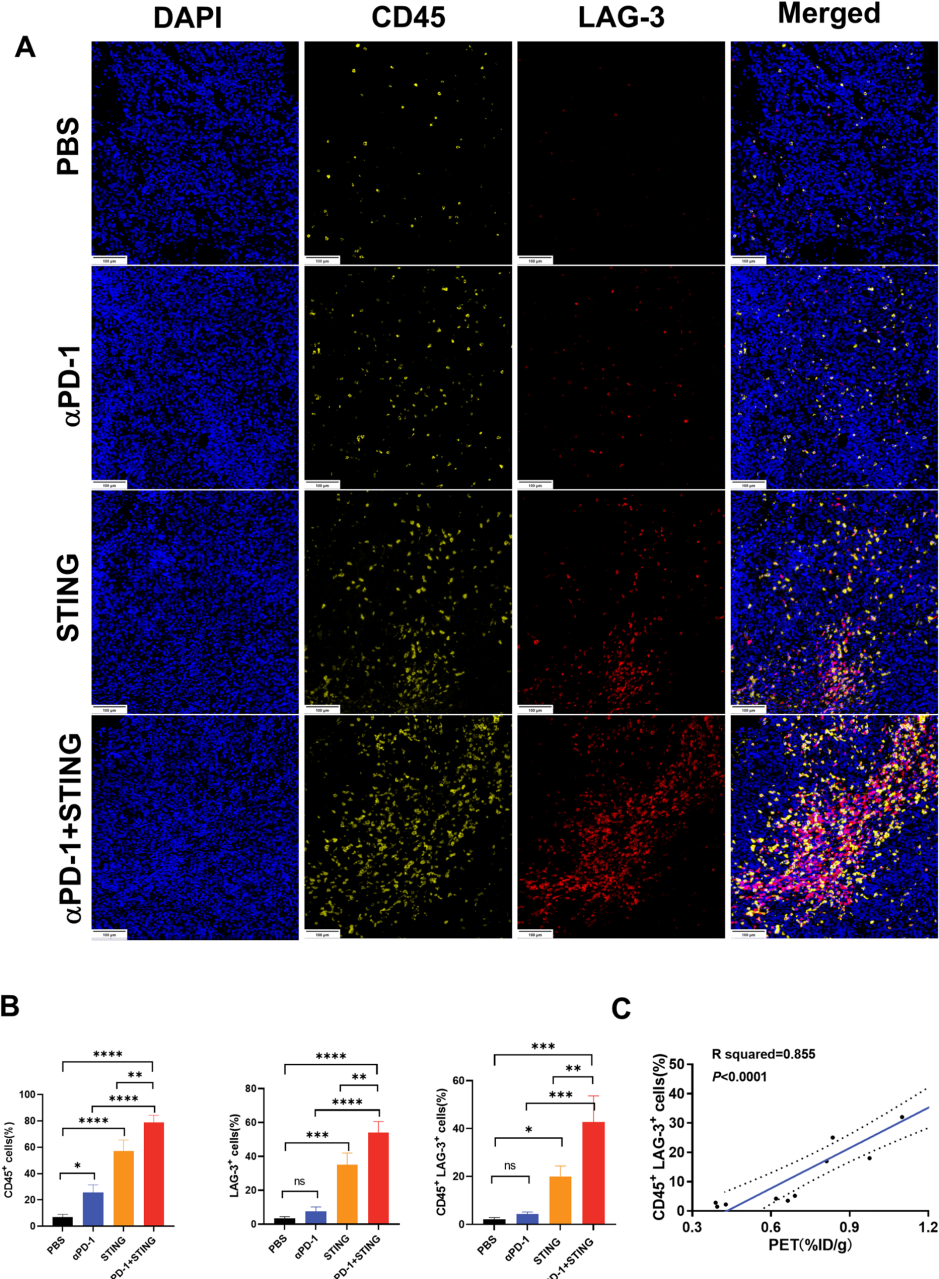
(**A**) Immunofluorescence images of LAG-3 and CD45 staining in the Hepa1-6 tumor at the treatment endpoint (DAPI: blue; CD45: yellow; LAG-3: red; Scale bar: 100 μm); (**B**) Quantitative analysis of LAG-3 and CD45 expression (*n* = 3); (**C**) Correlation analysis between CD45^+^ LAG-3^+^cells and ex vivo biodistribution (%ID/g) of [^68^Ga]Ga-NOTA-C25 at the treatment endpoint (*n* = 3). (*P < 0.05, **P < 0.01, *** *P* < 0.001, **** *P* < 0.0001)

**Fig. 4:**
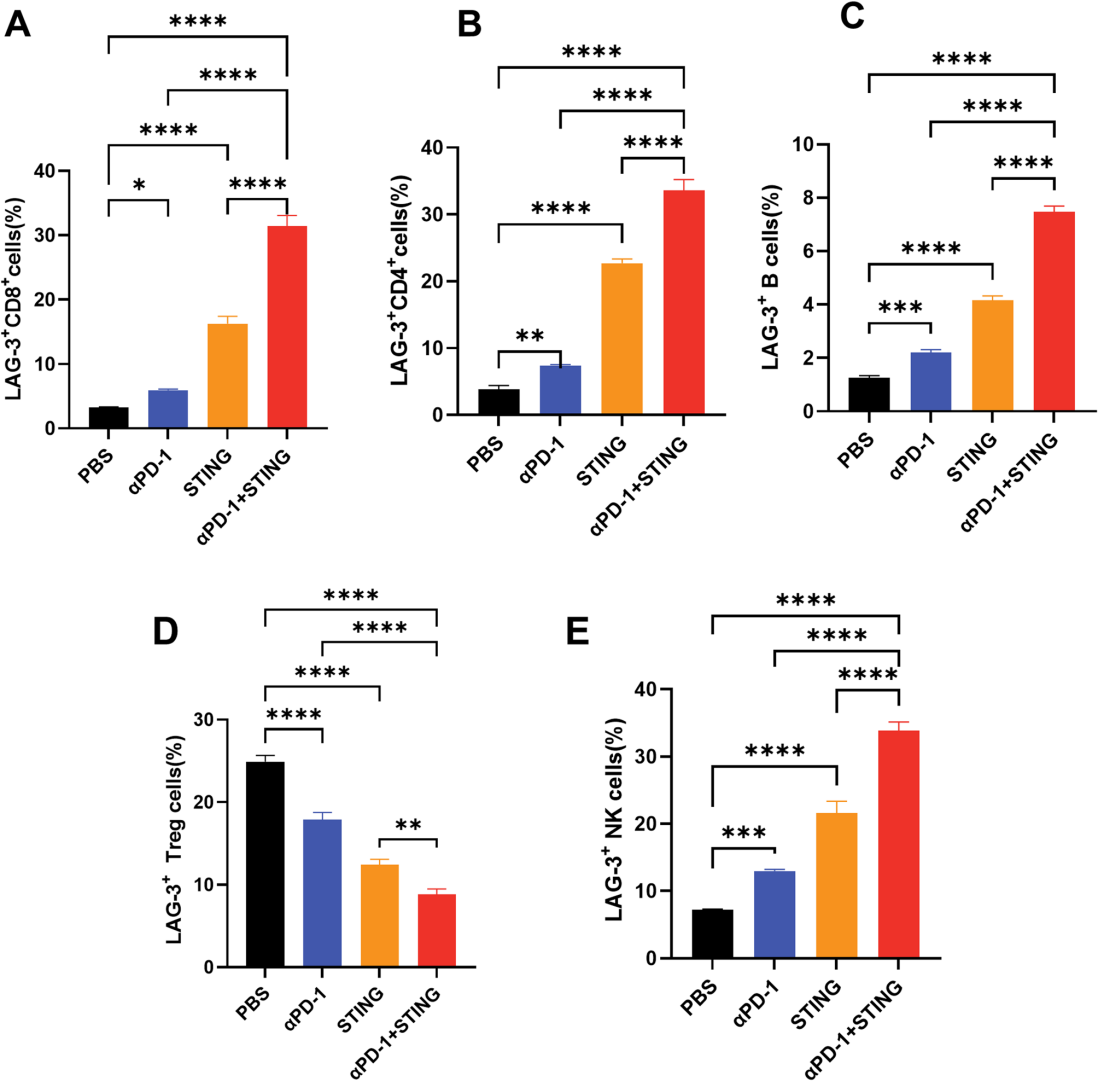
Flow cytometry analysis of LAG-3 expression on immune cell subsets in Hepa1-6 tumors at the treatment endpoint with anti-PD-1/STING agonist monotherapy and dual combination immunotherapy: (**A**) LAG-3^+^ CD8^+^ cells; (**B**) LAG-3^+^ CD4^+^ cells; (**C**) LAG-3^+^ B cells; (**D**) LAG-3^+^ Treg cells; (**E**) LAG-3^+^ NK cells. (*n* = 3). (*P < 0.05, **P < 0.01, *** *P* < 0.001, **** *P* < 0.0001)

Flow cytometry and immunofluorescence staining corroborated these observations, revealing an increase in LAG-3 expression on CD45^+^ TILs in the STING + αPD-1 group (Fig. 3A and B). Flow cytometry showed significantly elevated LAG-3^+^ TILs, except for Treg cells, in the combination immunotherapy group (STING agonist + anti-PD-1 mAb) compared to those of the PBS and monotherapy groups (Fig. 4, Fig.S4). Positive correlations were identified between [^68^Ga]Ga-NOTA-C25 uptake and the abundances of LAG-3^+^ CD8^+^ T cells, CD4^+^ T cells, B cells, and NK cells, while a negative correlation was observed with LAG-3^+^ Tregs (Fig. 3C, Fig.S5).

### Treatment efficacy of STING agonist/anti-PD-1 mAb monotherapy or dual immunotherapy

**Fig. 5:**
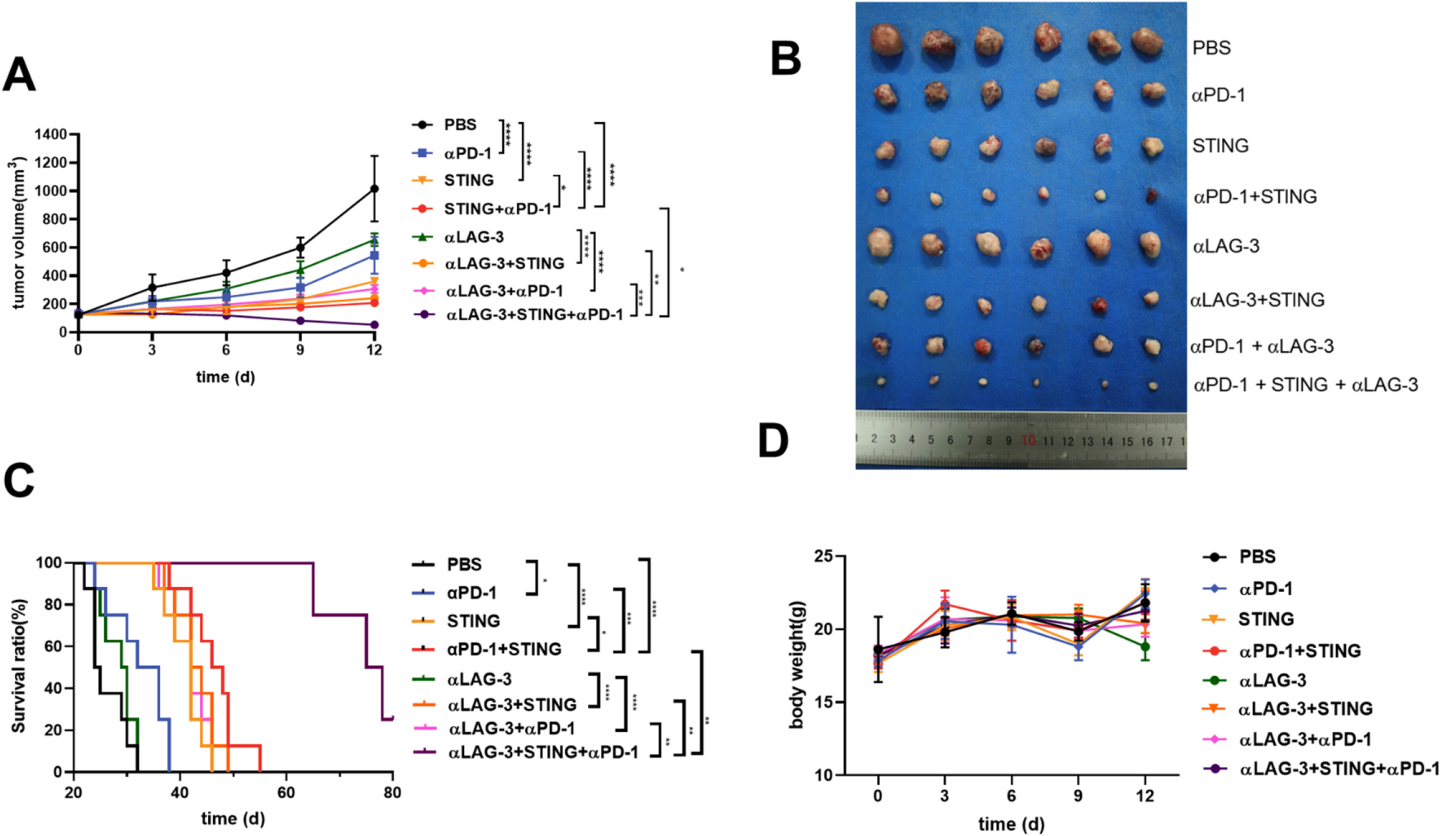
(**A**) Tumor growth curves of the Hepa1-6 tumor from different groups at different time points; (**B**) Photograph of tumor samples at the treatment endpoint (*n* = 6); (**C**) Survival analysis of Hepa1-6 tumor-bearing mice in different groups (*n* = 8); (**D**) Body weight changes in Hepa1-6 tumor-bearing mice in different groups (*n* = 6). (*P < 0.05, **P < 0.01, *** *P* < 0.001, **** *P* < 0.0001)

The antitumor efficacy of the STING agonist, anti-PD-1 mAb monotherapy, or dual combination immunotherapy was assessed. Tumor volumes in the αPD-1, STING, and STING + αPD-1 groups increased significantly slower compared to the PBS group. At the treatment endpoint, tumor volume in the STING + αPD-1 group (208 ± 23 mm^3^) was significantly lower than in any monotherapy group. Tumor volume in the STING group (361 ± 6 mm^3^) is less than in the αPD-1 (562 ± 138 mm^3^) and PBS groups (1016 ± 232 mm^3^) (Fig. 5A and B). The αPD-1 monotherapy showed a slight therapeutic effect and inhibited tumor growth. Survival times in Hepa1-6 tumor-bearing mice were 32 days (PBS), 38 days (αPD-1), 46 days (STING), and 55 days (STING + αPD-1) (Fig. 5C).

### Triple immunotherapy in murine models of HCC

**Fig. 6:**
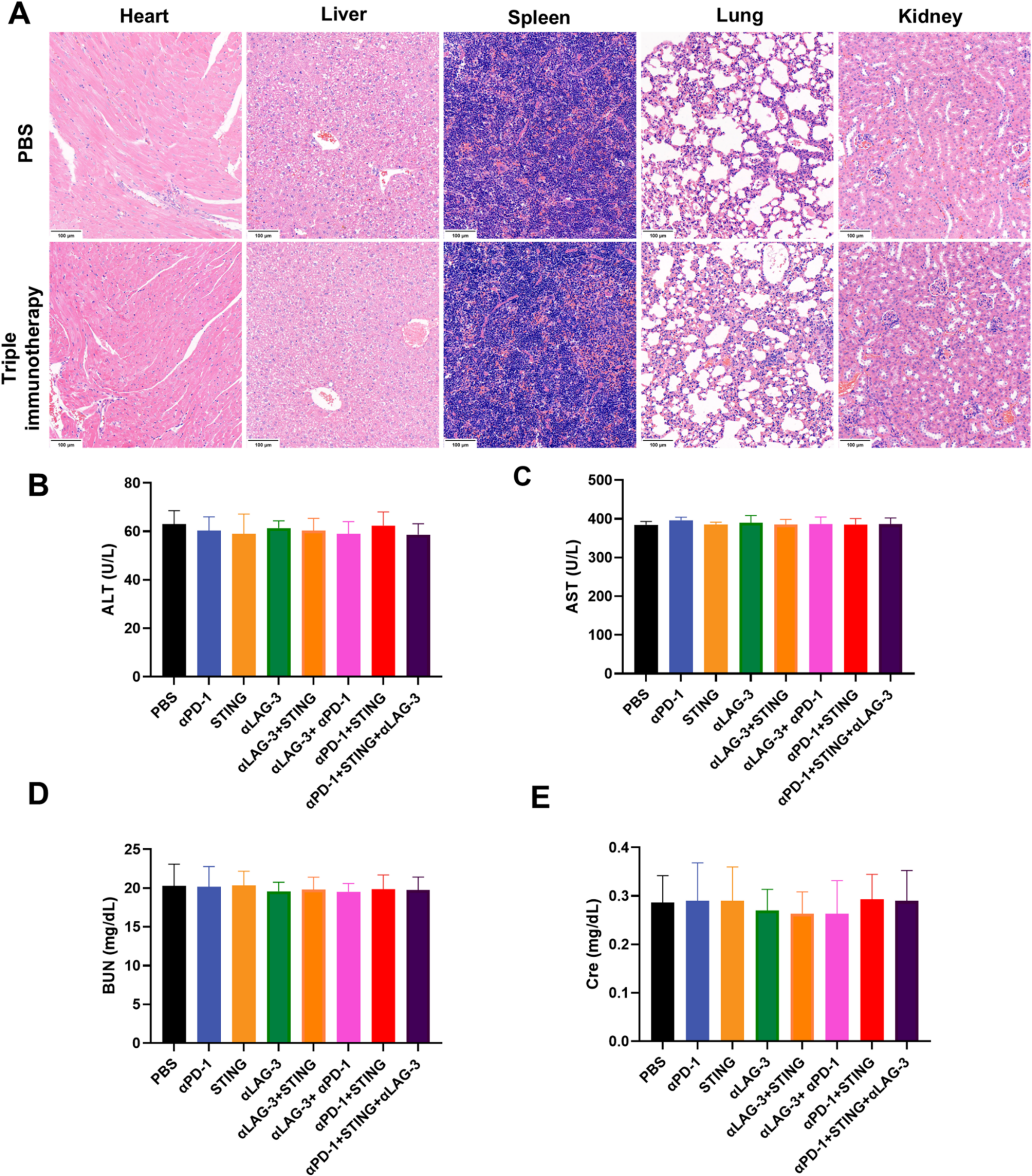
Safety assessment of triple immunotherapy after the treatment endpoint. (**A**) Hematoxylin-eosin (H&E) staining of organs (heart, liver, spleen, lung, kidney); Scale bar: 100 μm; (**B-E**) Liver and renal function markers, including alanine aminotransferase (ALT), aspartate aminotransferase (AST), blood urea nitrogen (BUN), and creatinine (Cre) (*n* = 3). Data are presented as mean ± SD

After further combination with anti-LAG-3 mAb and comparing it with dual combination immunotherapy, the triple immunotherapy significantly decreased tumor growth (53 ± 5 mm^3^ triple immunotherapy vs. 208 ± 23 mm^3^ STING + αPD-1 dual immunotherapy or 242 ± 17 mm^3^ STING + αLAG-3 dual immunotherapy or 307 ± 28 mm^3^ αPD-1 + αLAG-3 dual immunotherapy) (Fig. 5A and B). In Hepa1-6 tumor-bearing mice models, the survival time of the STING + αPD-1 dual immunotherapy, STING + αLAG-3 dual immunotherapy, and αPD-1 + αLAG-3 dual immunotherapy groups were 55, 49, and 49 days, respectively. In particular, the STING + αPD-1 + αLAG-3 (triple immunotherapy) group had not reached the endpoint even on day 80 (Fig. 5C). Compared to monotherapy or dual immunotherapy, triple immunotherapy significantly inhibited tumor growth and prolonged the survival time of mice. Importantly, no significant weight loss was observed in any of the treatment groups throughout the treatment period (Fig. 5D). Furthermore, the triple immunotherapy was well tolerated, with no observed toxicity in the major organs or abnormal levels of ALT, AST, BUN, or Cre in the blood samples (Fig. 6 and Fig S6). These findings suggest that anti-LAG-3 mAb improves the antitumor efficacy of STING + αPD-1 dual immunotherapy in mice without causing liver or kidney dysfunction.

### Triple immunotherapy modifies immune lymphocyte infiltration

**Fig. 7:**
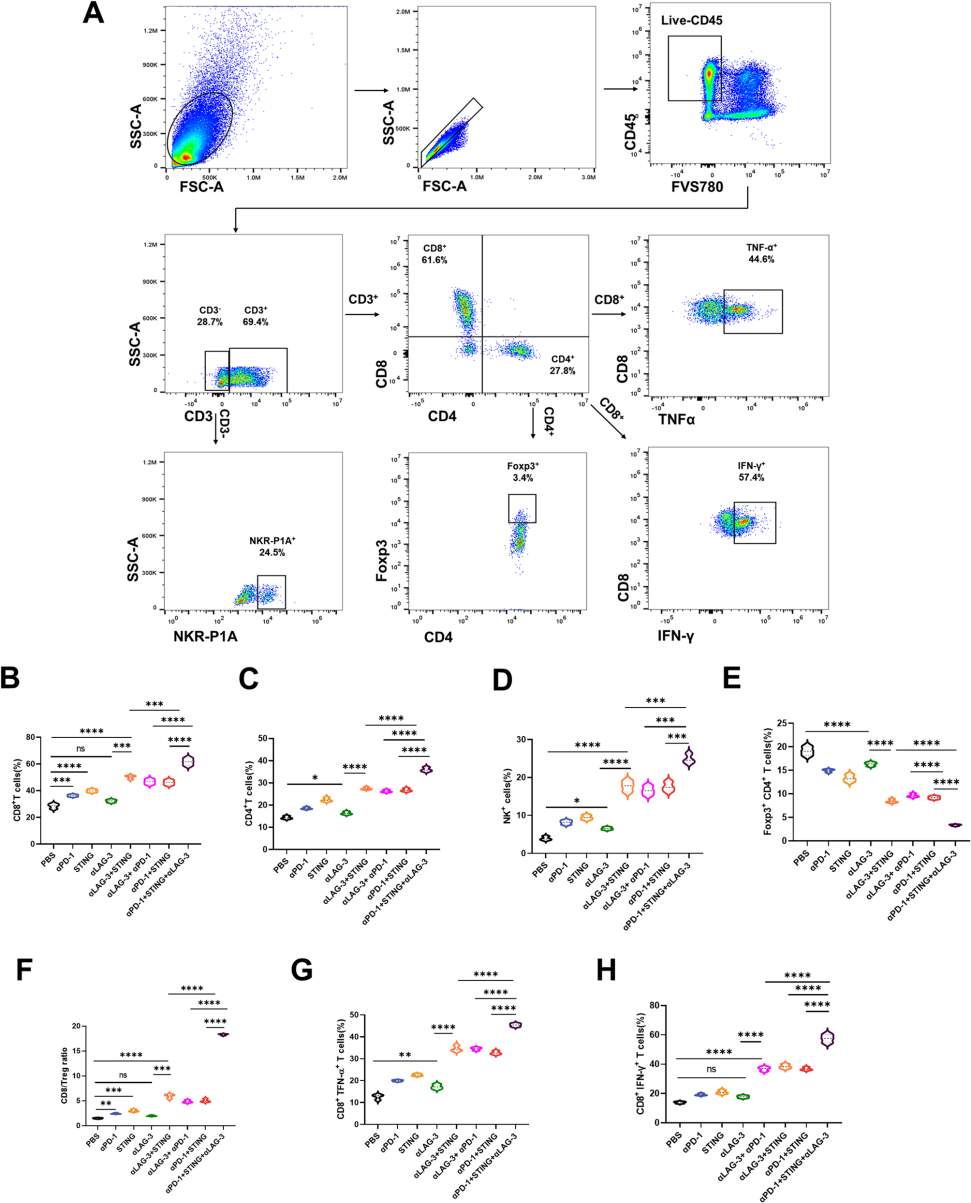
Triple immunotherapy improved antitumor immunity. (**A**) Representative dot plots of the morphological characteristics (SSC vs. FSC) of tumors subjected to triple immunotherapy. A schematic illustration of gating was used to analyze tumor-infiltrating lymphocytes stained with the corresponding antibodies for flow cytometry, including CD8^+^T, CD4^+^T, Treg cells, and NK cells. (**B-H**) Quantitative analysis of the percentages of CD8^+^T, CD4^+^T, Foxp3^+^CD4^+^ T, NK, CD8^+^ IFN-γ^+^ T, CD8^+^ TNF-α^+^ T cells, and CD8^+^ /Treg ratio in tumors after the treatment endpoint (*n* = 3). (*P < 0.05, **P < 0.01, *** *P* < 0.001, **** *P* < 0.0001)

Dual and triple immunotherapies induced changes in the tumor immune microenvironment compared to monotherapy, increasing the number of CD8^+^ T cells, CD4^+^ T cells, NK cells, and CD8^+^ /Treg ratio, while reducing Treg cells at the endpoint. The effect of triple immunotherapy was more significant than that of dual immunotherapy (Fig. 7A-F). Furthermore, triple immunotherapy significantly increased IFN-γ and TNF-α expression of CD8^+^ T cells compared to dual immunotherapy (Fig. 7A, G and H).

### Triple immunotherapy influences tumor cell proliferation and apoptosis

Hematoxylin-eosin (H&E) staining revealed extensive necrotic regions in Hepa1-6 tumors treated with triple immunotherapy compared to those in the dual immunotherapy, monotherapy, and PBS groups (Figure S7 A and B). The TUNEL assays showed a higher level of apoptosis in the triple immunotherapy group (Figure S8 A and B), accompanied by a significant reduction in the Ki67-positive rate in tumor cells (Figure S9 A and B). These results indicate that triple immunotherapy induces apoptosis more effectively and inhibits tumor cell proliferation more robustly than other treatment groups in Hepa1-6 tumor-bearing mice.

## Discussion

ICIs immunotherapy has emerged as a promising therapeutic approach. Several CTLA-4 and PD-1/PD-L1 mAbs have been approved by the FDA. However, the limited response rate due to primary or acquired resistance to ICIs remains a significant challenge. Therefore, reliable biomarkers are required for early treatment assessment, and novel combination immunotherapies are urgently needed [10]. A previous study developed a PET imaging probe targeting LAG-3, [^68^Ga]Ga-NOTA-C25, which allows the precise in vivo detection of LAG-3^+^ TILs [24].

In our study, we showed that [^68^Ga]Ga-NOTA-C25 PET imaging detected a significant increase in LAG-3^+^ TILs within Hepa1-6 tumors after combination immunotherapy with an anti-PD-1 mAb and a STING agonist. This indicates that the upregulation of LAG-3 on activated T cells in response to antigen stimulation reflects an ongoing immune response. Furthermore, triple immunotherapy combining anti-PD-1 mAb, a STING agonist, and anti-LAG-3 mAb markedly improved antitumor efficacy compared to monotherapy or dual therapy. Triple therapy reversed CD8^+^ T-cell dysfunction, induced tumor cell apoptosis, and inhibited tumor proliferation, resulting in reduced tumor growth and prolonged OS. In Hepa1-6 tumor-bearing mice, CD8^+^ TILs mediate pivotal therapeutic effects.

The limited effectiveness of PD-1/PD-L1 inhibitor monotherapy highlights the need for a more potent combination of immunotherapeutic strategies to significantly improve OS [3]. Two main approaches have emerged for combination immunotherapy: enhancing T-cell activation by blocking additional inhibitory checkpoints or activating stimulatory checkpoints and modulating the TME to support cytotoxic T-cell function or enhance antigen presentation [7]. Our study aligns with the first approach. We found that Hepa1-6 tumors, classified as “immunosuppressive,” showed moderate T-cell infiltration, where TME restricted immune cell activation and recruitment despite the absence of physical barriers [5, 32]. In these tumors, an alternative approach to overcome resistance to PD-1/PD-L1 directed immunotherapy may involve targeting additional inhibitory pathways within the TME [33]. The combination of LAG-3 inhibitors can mitigate the impairment of T-cell infiltration within the TME, thus enhancing its efficacy in supporting antitumor immune responses [34]. LAG-3 is expressed at a lower level in inactivated CD8^+^ T cells but is overexpressed in activated CD8^+^ TILs [35]. Therefore, LAG-3 is closely associated with cancer-specific T-cell dysfunction associated with PD-1 co-expression and could serve as a compensatory mechanism to overcome resistance to PD-1 blockade in patients [36–38].

In contrast, drug resistance can be attributed to intrinsic properties of the TME that hinder or curtail the efficacy of the immune response against tumors [39]. The absence of a response can be attributed to constraints on the activation or recruitment of T cells, presence of immunosuppressive cell subsets, production of immunosuppressive cytokines, and upregulation of immune checkpoints by cancer cells [40]. Activation of the cCAS/STING pathway can induce type I interferons production, which is known to facilitate antigen presentation, bolster B-cell antibody production, and improve the functionality of dendritic and T-cell populations [41]. STING agonists can significantly enhance the efficacy of immunotherapy in the treatment of solid tumors by promoting immune infiltration and orchestrating TME, particularly in the context of immunologically cold tumors [42–44]. Our results demonstrated that the combination of a STING agonist with anti-PD-1 mAb immunotherapy effectively transitioned tumors from an “immunosuppressive” to a “hot” phenotype. Combination immunotherapy of a STING agonist and anti-PD-1 mAb was found to be superior in inhibiting tumor growth and prolonged survival compared to monotherapy in tumors that showed moderate responsiveness to PD-1 blockade [28]. Importantly, these changes were detected early using [^68^Ga]Ga-NOTA-C25 PET imaging. In this study, we found that treatment with anti-LAG-3 mAb in the “hot Hepa1-6 tumors” could yield additional therapeutic effects, leading to a reduction in tumor volume and prolonged survival time compared with monotherapy or dual combination immunotherapy. In contrast, “immunosuppressive Hepa1-6 tumors’’ exhibit rapid progression and shorter survival times. The STING agonist significantly enhances the antitumor effects of a combined LAG-3/PD-1 blockade by stimulating the robust secretion of IFN-γ, TNF-α from CD8^+^, and CD4^+^ TILs, as well as reducing the number of immunosuppressive cells such as regulatory Treg cells. Compared to dual immunotherapy, triple immunotherapy not only increases the quantity, but also improves production capacity of IFN-γ in CD8^+^ TILs.

Current clinical trials on STING agonists are limited to intratumoral injections, thus restricting their broader application. Our study used the orally available STING agonist, MSA-2, which demonstrated effective tumor regression and durable antitumor immunity, showing significant potential for clinical translation and biosafety. The combination of MSA-2 with anti-PD-1 mAb and anti-LAG-3 mAb showed no signs of severe immunotoxicity in a murine model.

To the best of our knowledge, this is the first preclinical study to evaluate the therapeutic efficacy of triple immunotherapy with anti-PD-1 mAb, a STING agonist, and anti-LAG-3 mAb in a murine HCC model using LAG-3-targeting PET imaging. However, this study had some limitations. First, histological and flow cytometric analyses of LAG-3^+^ TILs and biological activity in the TDLN or spleen were not performed due to insufficient uptake of [^68^Ga]Ga-NOTA-C25. Second, triple immunotherapy effectively inhibited and attenuated tumor growth, maintaining it at a controlled level; however, complete tumor eradication was not achieved. More studies are needed to explore more potent therapeutic combinations, such as the combination of dual ICIs with tyrosine kinase inhibitor (TKI), radiotherapy, or the use of STING agonists in conjunction with mono ICI, TKI, or radiotherapy. Third, although triple immunotherapy comprising an anti-PD-1 mAb, STING agonist, and anti-LAG-3 mAb demonstrated adequate biosafety in murine HCC models, further investigations are warranted to evaluate its efficacy, potential toxicities, and optimal dosage in human subjects. Finally, the low baseline uptake of [^68^Ga]Ga-NOTA-C25 PET in the murine HCC models and the small tumor volume in the combination therapy group limited the accuracy of anti-LAG-3 mAb blockade on PET imaging. Additionally, it was not possible to determine whether the uptake changes were due to competitive binding of LAG-3 with [^68^Ga]Ga-NOTA-C25, or to treatment-induced effects. Therefore, we did not present the PET imaging results following anti-LAG-3 mAb blockade which is similar to previous studies ^31^.

## Conclusion

In conclusion, our study demonstrated that the combination of an anti-PD-1 mAb and a STING agonist markedly increased LAG-3^+^ TILs expression. Additionally, LAG-3-targeted PET imaging is an effective noninvasive method for visualizing the shift in the tumor immune phenotype from “immunosuppressive” to “hot”. Furthermore, when combined with an anti-LAG-3 mAb, triple immunotherapy was shown to be more effective than monotherapy or dual immunotherapy in inhibiting tumor growth.

## Supplementary Information

Below is the link to the electronic supplementary material.

Supplementary Material 1

## Data Availability

No datasets were generated or analysed during the current study.

## Abbreviations

HCC: Hepatocellular carcinoma
mAb: Monoclonal antibody
ICIs: Immune checkpoint inhibitors
FDA: Food and Drug Administration
PET: Positron emission tomography
NOTA: 1,4,7-triazacyclononane-1,4,7-triacetic acid
OS: Overall survival
LAG-3: Lymphocyte activation gene 3
CTLA-4: Cytotoxic T lymphocyte-associated antigen 4
PD-1: Programmed cell death protein 1
PD-L1: Programmed death ligand 1
Tregs: Regulatory T cells
NK cells: Natural killer cells
MHC-II: Major histocompatibility complex Class II
TILs: Tumor infiltrating lymphocytes
GMP-AMP: Cyclic guanosine monophosphate-adenosine monophosphate
cGAS/STING: Cyclic GMP–AMP synthase/stimulator of interferon genes
TME: Tumor microenvironment
BUN: Blood urea nitrogen
Cre: Creatinine
AST: Aspartate aminotransferase
ALT: Alanine aminotransferase
