# Supplementary material for: Combination immunotherapy targeting LAG-3, PD-1 and STING suppresses hepatocellular carcinoma as monitored by LAG-3 targeted PET imaging
Source: Biomark Res. 2025 Aug 12;13:102. doi: 10.1186/s40364-025-00820-z (PMC12344843; doi:10.1186/s40364-025-00820-z)
Supplement: Supplementary file 1 — Supplementary Material 1 [file 40364_2025_820_MOESM1_ESM.pdf]

Supporting Information

Combination immunotherapy targeting LAG-3, PD-1 and STING suppresses hepatocellular carcinoma as monitored by LAG-3 targeted PET imaging

Zhen Quan[1,2,3†], Yu Gao[3,4†], Bo Sun[1,2,3†], Yiwan Guo[1,2,3], Ziwei Jin[1,2,3], Na Hao[1,2,3], Dawei Jiang[3,4*], Chuansheng Zheng[1,2,3*], Xin Li[1,2,3*] Quan Chen[1,2,3*]

1. Department of Radiology, Union Hospital, Tongji Medical College, Huazhong University of Science and Technology, Jiefang Avenue #1277, Wuhan 430022, China.

2.Hubei Provincial Clinical Research Center for Precision Radiology & Interventional Medicine, Wuhan 430022, China

3.Hubei Key Laboratory of Molecular Imaging, Wuhan 430022, China.

4.Department of Nuclear Medicine, Union Hospital, Tongji Medical College, Huazhong University of Science and Technology, Wuhan, China

† Zhen Quan, Yu Gao and Bo Sun contributed equally to this work.

*Correspondence: Quan Chen

*Co-correspondence: Xin Li, Chuansheng Zheng, and Dawei Jiang

Address: No. 1277 Jiefang Avenue, Wuhan, Hubei. Tel: +86 85726114

Postcode:430022

#E-mail:

Zhen Quan: 2022xh0063@hust.edu.cn

Yu Gao: gyae862014@163.com

Bo Sun: sunbospace@163.com

Yiwan Guo: gyw19990107@163.com

Ziwei Jin: jzw688699@163.com

Na Hao: haonadoct@163.com

Dawei Jiang: daweijiang@hust.edu.cn

Chuansheng Zheng: hqzcsxh@sina.com

Xin Li: lxwsry2014@163.com

Quan Chen: chenquan1230@126.com

The PDF file includes:

Supplemental method

Figure S1 to S9

Table S1-2

Supplemental method

1.Immunofluorescence staining

Tumor tissues harvested from distinct experimental groups were fixed in 4% paraformaldehyde (PFA), followed by paraffin embedding. Serial sections (4 μm thickness) were prepared from paraffin-embedded blocks using a rotary microtome (Leica RM2016). Subsequently, sections were deparaffinized and subjected to antigen retrieval. To assess changes in LAG-3^{+} TILs following αPD-1/STING agonist treatment, the sections were blocked with 5% normal goat serum for 1 h at 25°C. After blocking, tumor sections were stained overnight at 4°C with primary antibodies: anti-CD45 antibody (Cat# ab8216, Abcam, Boston, MA) and anti-LAG-3 antibody (Cat# ab209238, Abcam, Boston, MA). For secondary staining, Goat Anti-Rabbit IgG H&L (Alexa Fluor® 647) (Cat# ab150079, Abcam, Boston, MA) and Goat Anti-Mouse IgG H&L (Alexa Fluor® 555) (Cat# ab150114, Abcam, Boston, MA) were used followed by a 1h incubation at room temperature. Cell nucleus staining was then performed using DAPI (Cat # C0065, Solarbio Beijing, China) for 15 min. To assess the effectiveness of the dual and triple immunotherapies, tissue sections were subjected to histological analysis using hematoxylin-eosin (H&E) staining, a TUNEL assay kit (Roche, Shanghai, China), and Ki67 monoclonal antibodies (Cat# ab15580, Abcam, Boston, MA). Immunofluorescence images were acquired using a Pannoramic MIDI II scanner (3DHistech), and analysis was performed using Case Viewer software (version 2.3.0).

2.Flow cytometry

At the endpoint of treatment, the mice were sacrificed and tumor tissues were collected. The tissues were then digested with collagenase IV (Solarbio, C8106) and DNase I (Solarbio, D8071) at 37°C for 30 min, followed by filtration through a 70 μm cell strainer. Single cells were obtained by grinding and filtering. Antibodies against LAG-3, CD45, CD3, CD4, CD8, CD19, NK1.1, IFN-γ, TNF-α, and Foxp3 were obtained from BD Biosciences. For intracellular cytokine staining with IFN-γ and TNF-α as well as Foxp3 staining using a Foxp3 Fixation and Permeabilization Kit (eBioscience), cells were permeabilized according to the manufacturer's protocols. Flow cytometry analysis was performed using a BD X20 instrument, and data analysis was conducted using FlowJo software (Tree Star, Ashland, OR).

3.Safety assessment

Major organs (heart, liver, spleen, lungs, and kidneys) were subjected to H&E staining. Peripheral

blood samples were collected from the retro-orbital sinus at the end of the treatment. Blood urea nitrogen (BUN), creatinine (Cre), aspartate aminotransferase (AST), and alanine aminotransferase (ALT) levels were measured to assess renal and hepatic functions.

Supplemental results

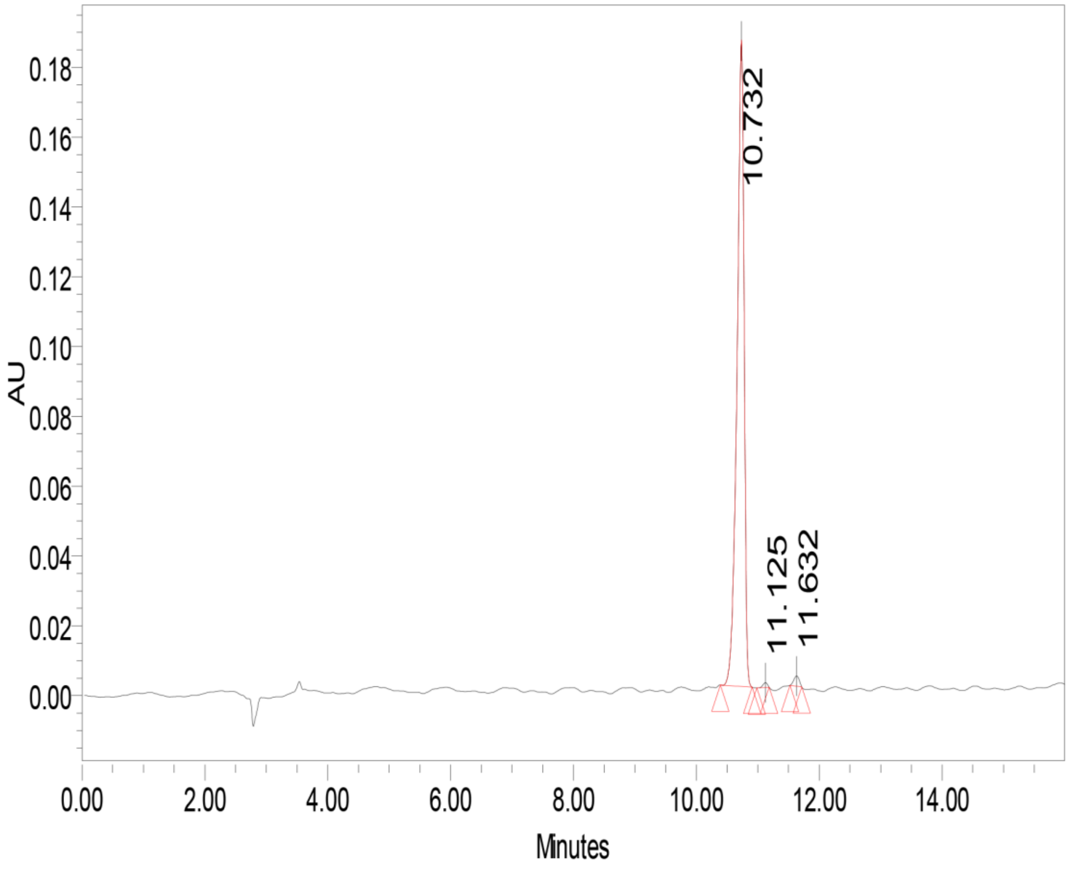

Fig.S1. High-performance liquid chromatography (HPLC) analysis of the precursor NOTA-C25 demonstrating a chemical purity greater than 98%, as certified by the manufacturer.

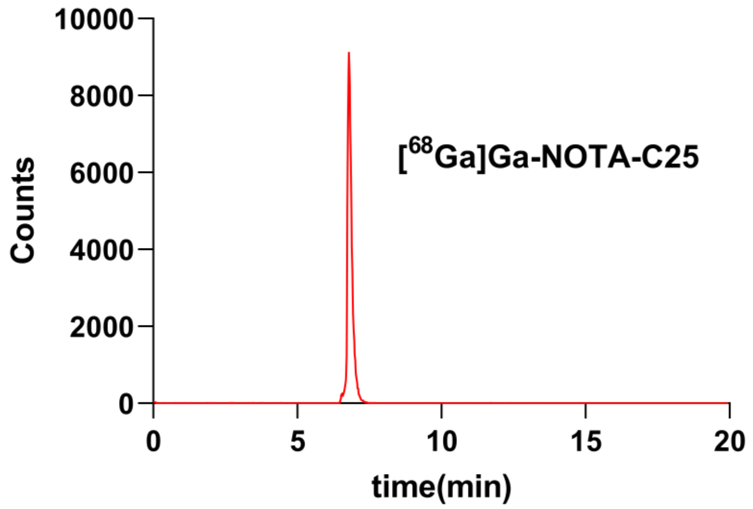

Fig.S2. [^{68}Ga]Ga-NOTA-C25 was successfully radiolabeled with high efficiency, achieving a radiochemical purity of over 99% as confirmed by radio-HPLC.

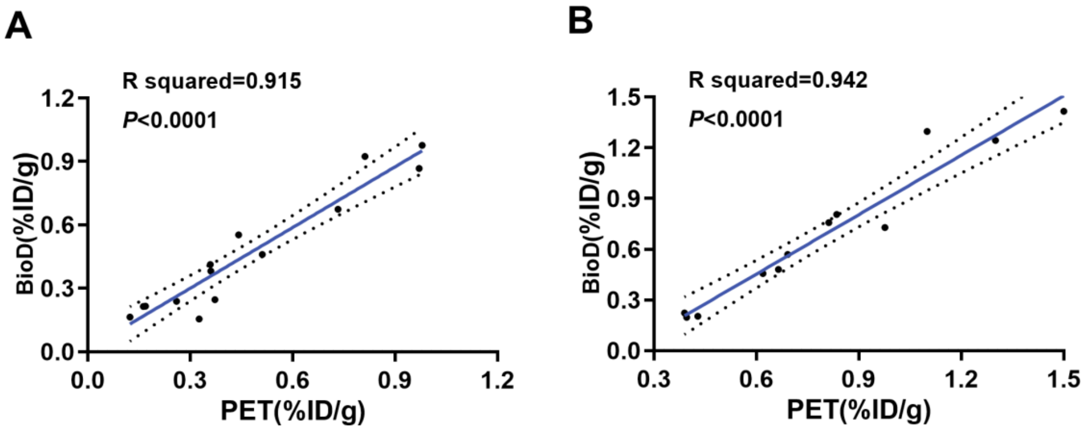

Fig.S3. (A) Correlation analysis of PET uptake and ex vivo biodistribution (%ID/g) of [^{68}Ga]Ga NOTA-C25 in Hepa1-6 tumor in the non-blocking and blocking study. (B) Correlation analysis of PET uptake and ex vivo biodistribution (%ID/g) of [^{68}Ga]Ga NOTA-C25 in Hepa1-6 tumor on day 13 after anti-PD-1/STING agonist monotherapy and dual combination therapy.

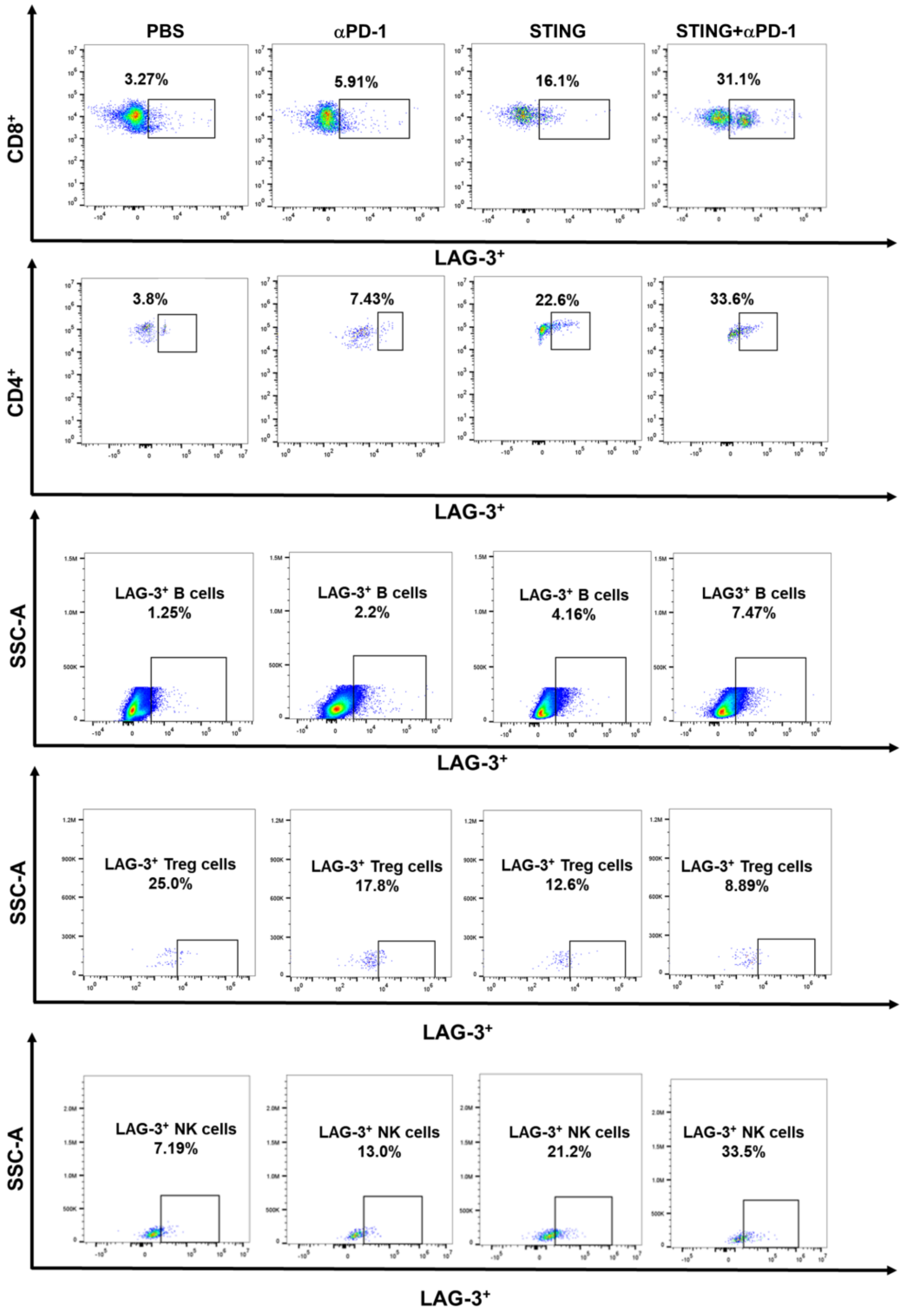

Fig.S4. Flow cytometry was used to detect LAG-3 expression in different immune cell subsets in Hepa1-6 tumors treated with anti-PD-1/STING agonist monotherapy and dual combination immunotherapy at the endpoint of treatment.

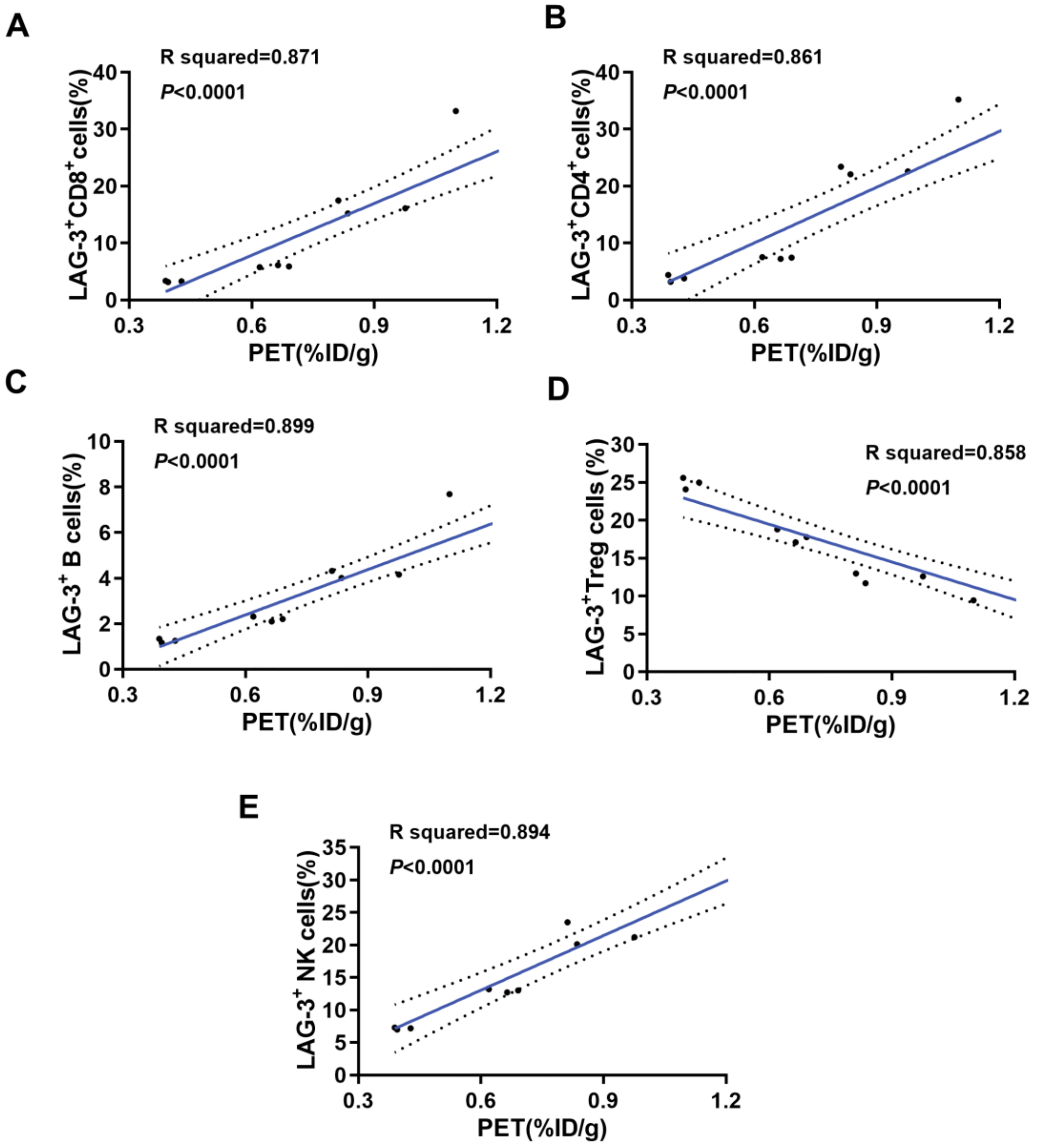

Fig. S5. (A) Correlation analysis of LAG-3 on different immune cell subsets in Hepa1-6 tumors of anti-PD-1/STING agonist monotherapy and dual combination immunotherapy as analyzed by flow cytometry and PET uptake (%ID/g) of [^{68}Ga]Ga NOTA-C25 at the endpoint of treatment. (A) LAG-3^{+}CD8^{+} cells; (B) LAG-3^{+}CD4^{+} cells; (C) LAG-3^{+}B cells; (D) LAG-3^{+}Treg cells;(E) LAG-3^{+}NK cells.

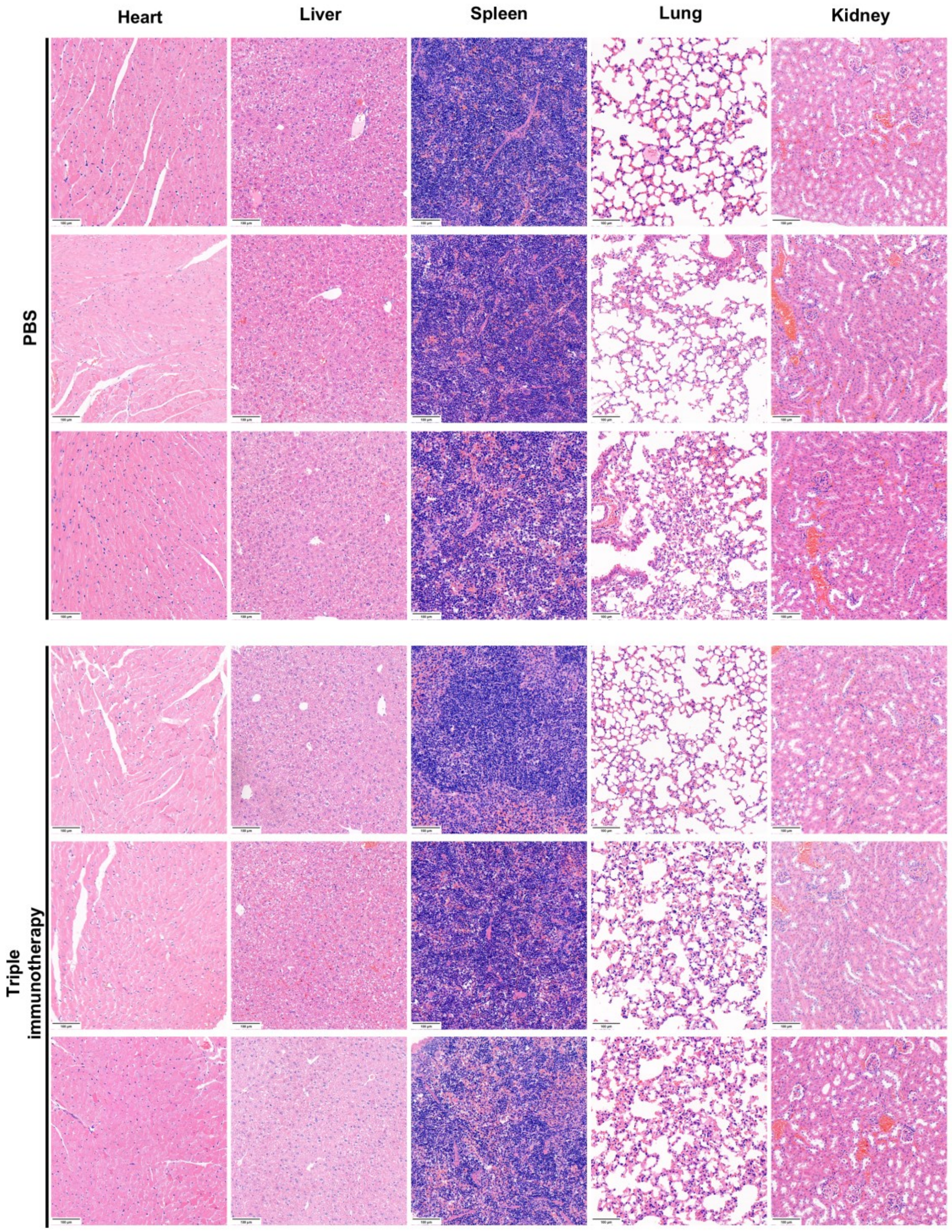

Fig. S6.Hematoxylin-eosin (H&E) staining of organs (heart, liver, spleen, lung, kidney); Scale bar: 100 μm.

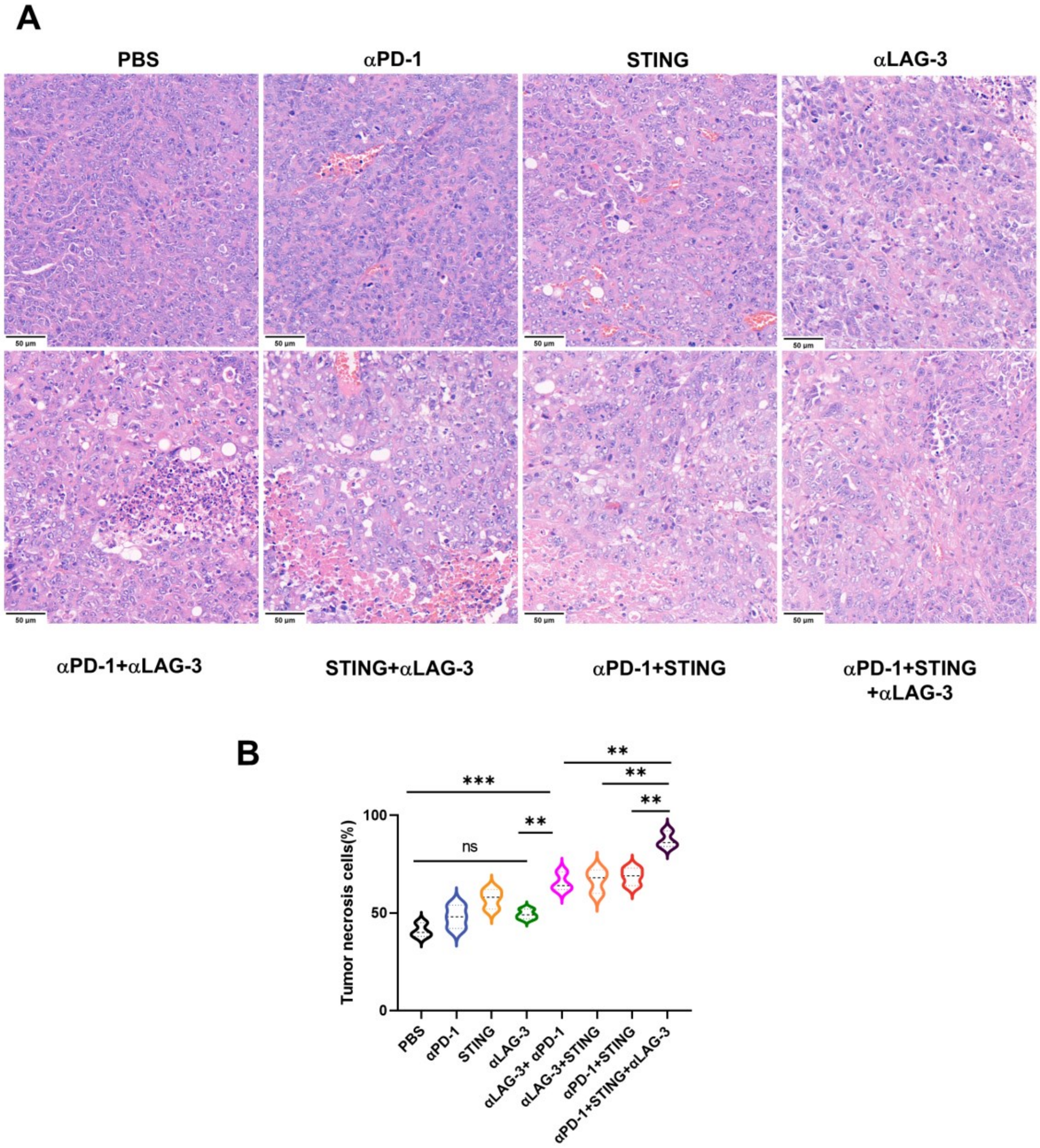

Fig. S7. (A) Representative H&E in Hepa1-6 tumor sections (scale bar, 50 μm). (B) Quantitative analysis of tumor necrosis cells in different immunotherapy groups at the endpoint of treatment. (n = 3 per group, *$P < 0.05$, **$P < 0.01$, *** $P < 0.001$, **** $P < 0.0001$)

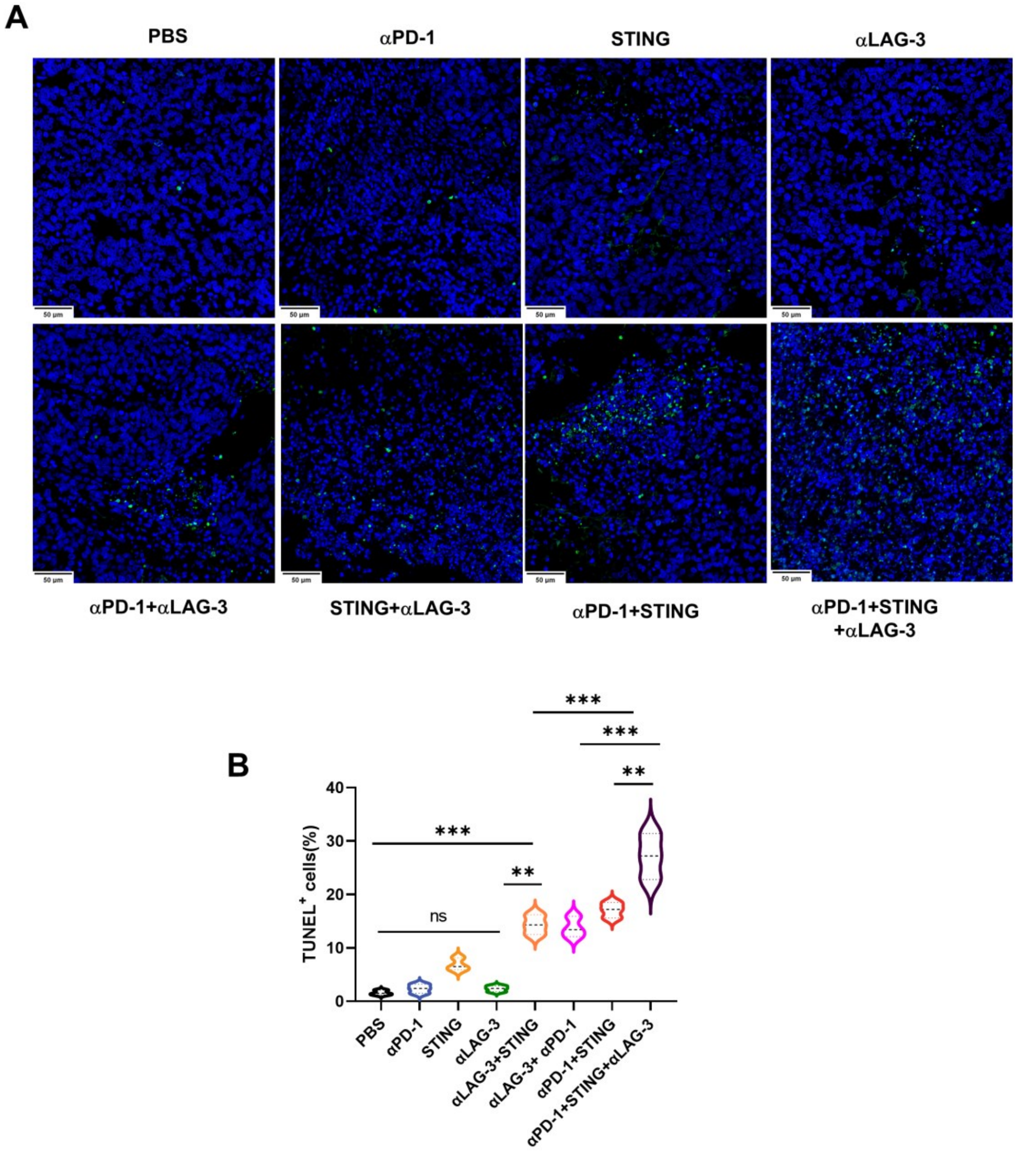

Fig. S8. (A) Representative immunofluorescence staining (TUNEL) in Hepa1-6 tumor sections to assess tumor apoptosis (scale bar, 50 μm). (B) Quantitative analysis of $TUNEL^+$ tumor cells in different immunotherapy groups at the endpoint of treatment. (n = 3 per group, *$P < 0.05$, **$P < 0.01$, *** $P < 0.001$, **** $P < 0.0001$).

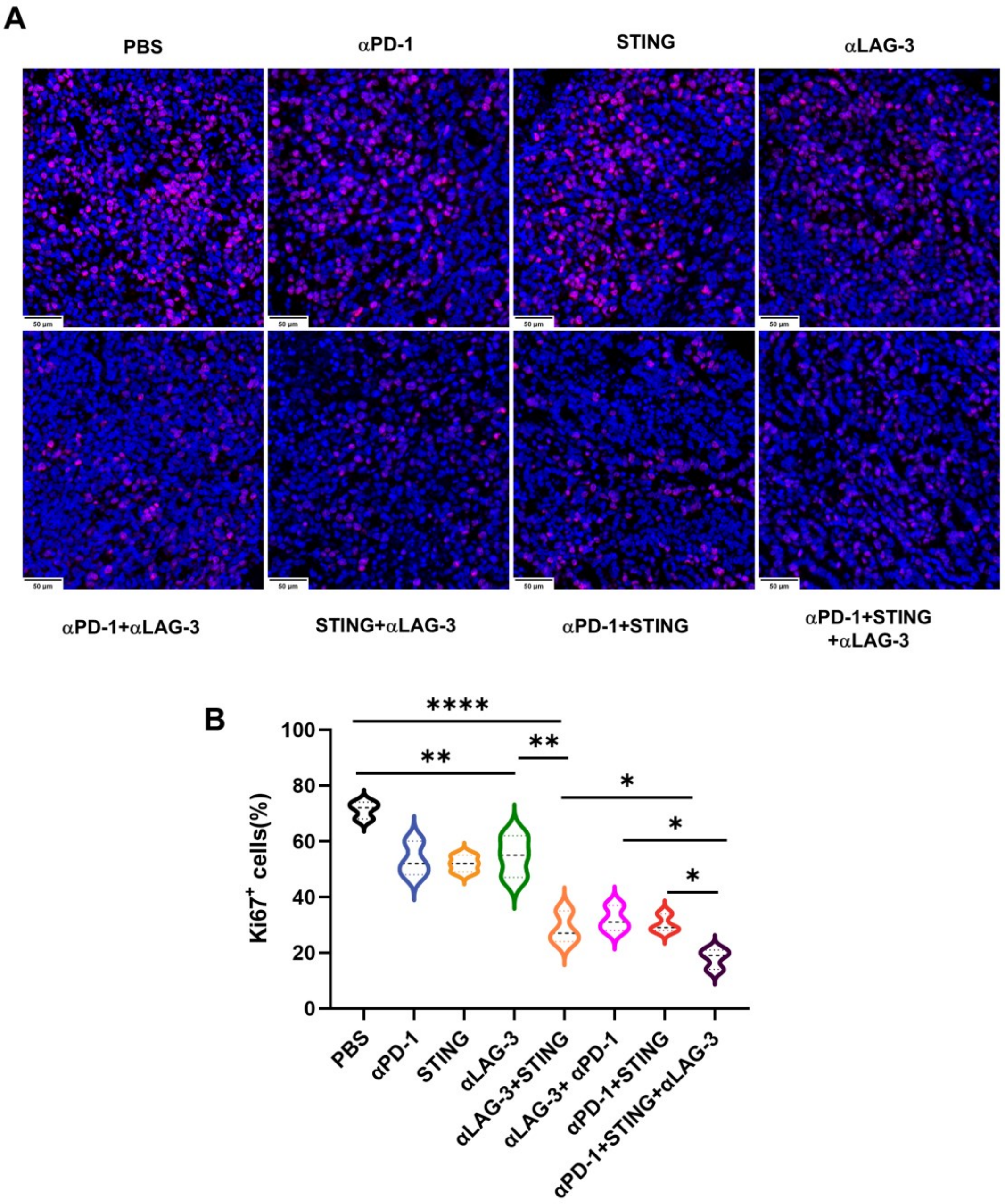

Fig. S9. (A) Representative immunofluorescence staining (Ki67) in Hepa1-6 tumor sections to assess tumor proliferation (scale bar, 50 μm). (B) Quantitative analysis of tumor Ki67^{+} cells in different immunotherapy groups at the endpoint of treatment. (n = 3 per group, *$P < 0.05$, **$P < 0.01$, *** $P < 0.001$, **** $P < 0.0001$).

Table S1. Ex vivo biodistribution of [^{68}Ga]Ga-NOTA-C25 in Hepa1-6 tumor-bearing mouse (n=3).

	Hepa1-6			
	30min	60min	60min blocked	120min
Blood	0.68±0.08	0.31±0.04	0.38±0.06	0.13±0.04
Skin	0.51±0.03	0.34±0.11	0.40±0.09	0.29±0.06
Muscle	0.21±0.05	0.05±0.01	0.06±0.01	0.05±0.01
Femur	0.31±0.08	0.15±0.05	0.13±0.04	0.16±0.05
Tumor	0.86±0.13	0.48±0.07	0.21±0.04	0.25±0.10
TDLN	0.29±0.06	0.23±0.03	0.15±0.03	0.23±0.05
Liver	0.33±0.09	0.19±0.05	0.19±0.02	0.09±0.05
Spleen	0.44±0.14	0.23±0.04	0.12±0.01	0.15±0.05
Kidney	9.10±1.85	7.90±1.24	8.01±0.95	5.34±1.25
Stomach	0.48±0.09	0.18±0.06	0.14±0.04	0.12±0.02
Intestine	0.60±0.10	0.33±0.07	0.15±0.03	0.19±0.03
Heart	0.40±0.10	0.11±0.01	0.10±0.03	0.12±0.02
Lung	0.60±0.08	0.20±0.05	0.16±0.02	0.16±0.05
Brain	0.07±0.02	0.02±0.00	0.01±0.00	0.02±0.00

Table S2. Ex vivo biodistribution of [^{68}Ga]Ga-NOTA-C25 in Hepa1-6 tumor-bearing mice after immunotherapy at the time endpoint (n=3).

	Hepa1-6			
	PBS	αPD-1	STING	STING+αPD-1
Blood	0.16±0.01	0.13±0.02	0.25±0.06	0.27±0.02
Skin	0.29±0.03	0.24±0.04	0.33±0.10	0.43±0.05
Muscle	0.19±0.05	0.13±0.03	0.20±0.02	0.22±0.04
Femur	0.23±0.06	0.28±0.03	0.30±0.04	0.31±0.02
Tumor	0.21±0.01	0.50±0.06	0.76±0.04	1.32±0.09
TDLN	0.25±0.06	0.34±0.03	0.41±0.04	0.48±0.11
Liver	0.14±0.03	0.17±0.03	0.19±0.06	0.25±0.04
Spleen	0.18±0.04	0.25±0.01	0.42±0.02	0.50±0.04
Kidney	6.65±0.33	7.44±0.17	7.61±0.44	7.82±0.40
Stomach	0.19±0.04	0.29±0.04	0.28±0.05	0.29±0.05
Intestine	0.14±0.03	0.12±0.03	0.22±0.02	0.21±0.07
Heart	0.11±0.01	0.12±0.02	0.10±0.02	0.17±0.03
Lung	0.21±0.06	0.21±0.13	0.30±0.08	0.24±0.06
Brain	0.02±0.00	0.01±0.00	0.01±0.00	0.02±0.01
